# Patients with uterine leiomyoma exhibit a high incidence but low mortality rate for breast cancer

**DOI:** 10.18632/oncotarget.16520

**Published:** 2017-03-23

**Authors:** Te-Chun Shen, Te-Chun Hsia, Chieh-Lun Hsiao, Cheng-Li Lin, Chih-Yi Yang, Khay-Seng Soh, Liang-Chih Liu, Wen-Shin Chang, Chia-Wen Tsai, Da-Tian Bau

**Affiliations:** ^1^ Graduate Institute of Clinical Medical Science, College of Medicine, China Medical University, Taichung, Taiwan; ^2^ Division of Pulmonary and Critical Care Medicine, Department of Internal Medicine, China Medical University Hospital, Taichung, Taiwan; ^3^ Terry Fox Cancer Research Laboratory, China Medical University Hospital, Taichung, Taiwan; ^4^ Management Office for Health Data, China Medical University Hospital, Taichung, Taiwan; ^5^ Department of Obstetrics and Gynecology, China Medical University Hospital, Taichung, Taiwan; ^6^ Department of Breast Surgery, China Medical University Hospital, Taichung, Taiwan; ^7^ Department of Bioinformatics and Medical Engineering, Asia University, Taichung, Taiwan

**Keywords:** breast cancer, uterine leiomyoma, cohort study

## Abstract

The association of uterine leiomyoma with increased risk of breast cancer is controversial. Therefore, we used the National Health Insurance Research Database of Taiwan to examine breast cancer incidence and mortality among Asian patients with and without uterine leiomyoma. We compared breast cancer incidence and mortality between 22,001 newly diagnosed uterine leiomyoma patients and 85,356 individuals without uterine leiomyoma matched by age and date of diagnosis. Adjusted hazard ratios for breast cancer were estimated using the Cox model. The incidence of breast cancer was 35% higher in the uterine leiomyoma group than the leiomyoma-free group (1.65 vs. 1.22 per 1,000 individuals, *p* < 0.001), with an adjusted hazard ratio of 1.31 (95% confidence interval = 1.13−1.52). Interestingly, overall mortality was lower (4.12%) in the uterine leiomyoma group (mean followed time, 3.59 ± 2.70 years) than the leiomyoma-free group (8.78%; mean followed time, 3.54 ± 2.67 years) at the endpoint of the study (p <0.05). These findings indicate the incidence of breast cancer is higher in patients with uterine leiomyoma than in those without it, but overall mortality from breast cancer was lower in the patients with uterine leiomyoma.

## INTRODUCTION

Uterine leiomyoma is a highly prevalent, benign smooth muscle tumor of the uterus. It is classified based on the location in the uterus as either intramural, submucosal or subserosal types [1]. It occurs in more than 50% of all women of reproductive age and nearly 30% of women seek treatment after experiencing troublesome symptoms [1, 2]. Hormonal disturbances and genetic alterations are postulated to contribute to the development of uterine leiomyoma, although their precise functions are not completely understood [3]. In some cases, uterine leiomyoma is associated with other malignancies, especially gynecological cancers such as ovarian and endometrial cancers [4–6].

Breast cancer is the most common type of cancer in women and ranks second in cancer death among women worldwide (http://www.who.int/cancer/en). Nearly 231,840 new cases of invasive breast cancer and 40,290 breast cancer deaths were reported among US women in 2015 [7]. According to the Taiwanese government (http://www.hpa.gov.tw), the incidence of female breast cancer was 86.9 for every 100,000 women in 2011. Age, reproductive factors, personal or family history of breast disease, genetic predisposition, and environmental factors are associated with the development of breast cancer [8]. Further, gynecological diseases such as polycystic ovary syndrome [9], endometriosis [10], and adenomyosis [10] are also associated with an increased risk of breast cancer.

The association of uterine leiomyoma with increased risk of breast cancer is controversial. Grosse *et al* reported that autopsies of individuals with uterine leiomyoma were most frequently associated with breast cancer [11]. Lindegård reported significant association between uterine leiomyoma and non-fatal breast cancer after investigating 162,449 women in Gothenburg [12]. In a recent case-control study in Taiwan, Chuang *et al* reported that prior history of uterine leiomyoma was associated with the development of breast cancer [odds ratio (OR): 1.42, 95% confidence interval (CI) = 1.24−1.63] [13]. On the contrary, a large-scale study of a population of African-American women reported that previous history of uterine leiomyoma was not significantly associated with subsequent breast cancer [14].

The National Health Insurance Research Database in Taiwan is a nationwide database that has previously contributed reliable data to various studies, including those focused on uterine leiomyoma and breast cancer [15–18]. In this study, we investigated if uterine leiomyoma increased breast cancer risk by comparing the incidence and mortality of breast cancer between patients with uterine leiomyoma and the general population using the data from National Health Insurance Research Database.

## RESULTS

### Comparison of general features between patients with or without uterine leiomyoma

This study included 22,001 patients in the uterine leiomyoma group and 85,356 individuals in the leiomyoma-free group (Table 1). Nearly 65% patients analyzed in this study were less than 45 years of age (mean age: 41 years). Further, nearly 82.1% in the uterine leiomyoma group and 91.6% in the leiomyoma-free group had not received any mammogram screening at the time of our study. Also, compared with the leiomyoma-free group, patients in the uterine leiomyoma group demonstrated more hypertension, hyperlipidemia, benign breast tumor, menopausal and postmenopausal disorders, obesity, and infertility. In addition, compared to the leiomyoma-free group, patients in the uterine leiomyoma group used more estrogen (6.32% vs 1.94%), progesterone (6.16% vs 2.32%) or both medications (2.64% vs 1.08%). The mean follow-up times in the uterine leiomyoma group and leiomyoma-free group were 6.71 (SD = 3.28) and 6.77 (SD = 3.17) years, respectively.

**Table 1:** Age, comorbidity, mammogram screening, and medication in individuals with and without uterine leiomyoma

Variable	Uterine leiomyoma		*p*-value
	No	Yes	
	N (%)	N (%)	
**Age group**			0.97
20−39	31688 (37.1)	8157 (37.1)	
40−44	24092 (28.2)	6201 (28.2)	
≥ 45	29576 (34.7)	7643 (34.7)	
Mean ± SD^†^	41.3 ± 8.28	41.9 ± 7.43	< 0.001
**Mammogram screening**			< 0.001
0	78176 (91.6)	18058 (82.1)	
1	5008 (5.87)	2406 (10.9)	
2	1261 (1.48)	824 (3.75)	
≥ 3	911 (1.07)	713 (3.24)	
**Comorbidity**			
Hypertension	8162 (9.56)	2475 (11.3)	< 0.001
Diabetes	2254 (2.64)	538 (2.45)	0.10
Hyperlipidemia	6468 (7.58)	2257 (10.3)	< 0.001
TUD	232 (0.27)	69 (0.31)	0.30
ARD	756 (0.89)	206 (0.94)	0.48
BBT	3519 (4.12)	1798 (8.17)	< 0.001
MPD	7554 (8.85)	2708 (12.3)	< 0.001
Obesity	1256 (1.47)	409 (1.86)	< 0.001
Infertility	2227 (2.61)	968 (4.40)	< 0.001
**Medication**			
Estrogen	1654 (1.94)	1391 (6.32)	< 0.001
Progesterone	1976 (2.32)	1355 (6.16)	< 0.001
Both	921 (1.08)	581 (2.64)	< 0.001

Chi-square test; ^†^ *t*-test; TUD, tobacco use disorders; ARD, alcohol-related diseases; BBT, benign breast tumor; MPD, menopausal and postmenopausal disorders.

### Breast cancer risk in patients with or without uterine leiomyoma

**Figure 1:**
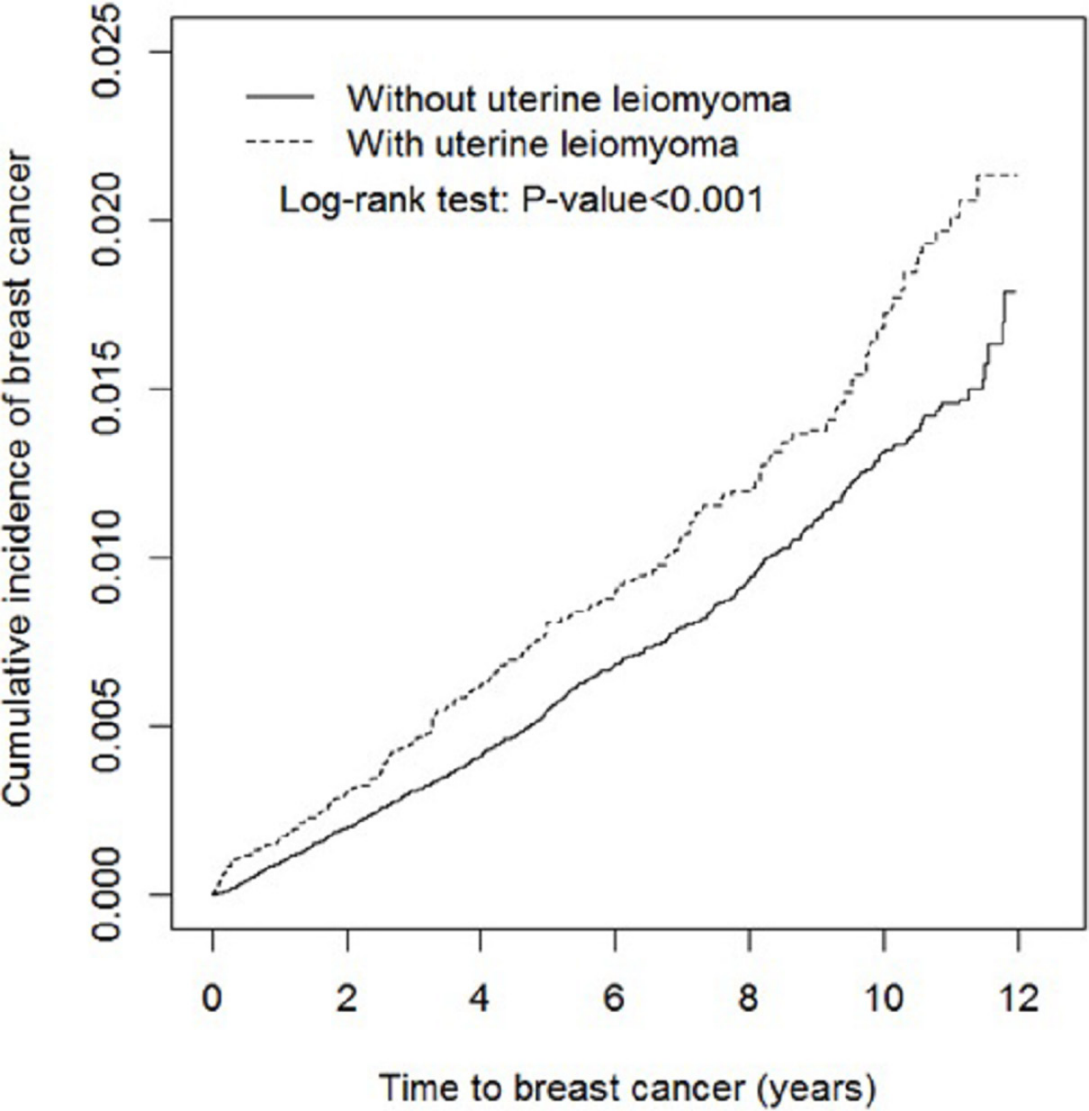
Cumulative incidence of breast cancer for individuals with (dashed line) and without (solid line) uterine leiomyoma

The overall incidence of breast cancer was 1.65 and 1.22 per 1000 women in the uterine leiomyoma group and the leiomyoma-free group, respectively (Table 2). The results showed that the cumulative incidence of breast cancer was significantly higher in the uterine leiomyoma group than the leiomyoma-free group (log-rank test *p* < 0.001) at the end of the follow-up period (Figure 1).

**Table 2:** Incidences and hazard ratios for breast cancer and breast cancer-associated factors

Variable	Event	PY	Rate#	Crude HR (95% CI)	Adjusted HR^†^ (95% CI)
**Uterine leiomyoma**					
No	706	577858	1.22	1.00	1.00
Yes	243	147581	1.65	1.35 (1.16, 1.56)***	1.31(1.13, 1.52)***
**Age group**					
20−39	196	265662	0.74	1.00	1.00
40−44	333	209478	1.59	2.15(1.80, 2.56)***	2.11(1.77, 2.52)***
≥ 45	420	250298	1.68	2.27(1.92, 2.69)***	2.19(1.82, 2.63)***
**Mammogram screening**					
0	824	631905	1.30	1.00	1.00
1	89	60489	1.47	1.09 (0.88, 1.36)	-
2	20	17930	1.12	0.82 (0.53, 1.28)	-
≥ 3	16	15114	1.06	0.77 (0.47, 1.26)	-
**Comorbidity**					
Hypertension					
No	826	651932	1.27	1.00	1.00
Yes	123	73507	1.67	1.32 (1.09, 1.59)**	1.05(0.85, 1.29)
Diabetes					
No	916	707931	1.29	1.00	1.00
Yes	33	17507	1.88	1.48 (1.05, 2.10)*	1.26(0.87, 1.81)
**Hyperlipidemia**					
No	854	665936	1.28	1.00	1.00
Yes	95	59503	1.60	1.25 (1.01, 1.54)*	0.93(0.74, 1.17)
TUD					
No	947	724433	1.31	1.00	1.00
Yes	2	1006	1.99	1.71(0.43, 6.85)	-
ARD					
No	940	720102	1.31	1.00	1.00
Yes	9	5336	1.69	1.34(0.69, 2.58)	-
BBT					
No	849	693056	1.23	1.00	1.00
Yes	100	32383	3.09	2.58 (2.10, 3.18)***	2.39(1.94, 2.95)***
MPD					
No	817	652027	1.25	1.00	1.00
Yes	132	73411	1.80	1.43 (1.19, 1.72)***	1.08(0.88, 1.32)
Obesity					
No	938	716310	1.31	1.00	1.00
Yes	11	9128	1.21	0.96 (0.53, 1.74)	-
Infertility					
No	939	708639	1.33	1.00	1.00
Yes	10	16799	0.60	0.47 (0.25, 0.88)*	0.60(0.32, 1.12)
**Medication**					
Estrogen					
No	916	697353	1.31	1.00	1.00
Yes	33	28086	1.17	0.84(0.60, 1.20)	-
Progesterone					
No	922	695991	1.32	1.00	1.00
Yes	27	29448	0.92	0.66(0.45, 0.97)*	0.53(0.36, 0.77)**

ARD, alcohol-related diseases; BBT, benign breast tumor; CI, confidence interval; HR, hazard ratio; MPD, menopausal and postmenopausal disorders; PY, person-years; TUD, tobacco use disorders;

^#^Incidence rate per 1,000 person-years;

^†^ Multivariable analysis including age, comorbidities of hypertension, diabetes, hyperlipidemia, benign breast tumor, menopausal and postmenopausal disorders and infertility, and medication of progesterone;

*p < 0.05, **p < 0.01, ***p < 0.001.

Table 2 shows the potential factors that affected the incidence rates and hazard ratios for breast cancer. After adjustment for age, comorbidities of hypertension, diabetes, hyperlipidemia, benign breast tumor, menopausal and postmenopausal disorders, infertility, and medication of progesterone, the uterine leiomyoma group was associated with higher risk of breast cancer than the leiomyoma-free group [adjusted hazard ratio (HR) = 1.31, 95% CI = 1.13−1.52]. Compared to individuals aged between 20 and 39 years, the risk of breast cancer was 2.11-fold higher in individuals between 40 and 45 years (95% CI = 1.77−2.52), and 2.19-fold higher in those aged ≥ 45 years (95% CI = 1.82−2.63). The risk of developing breast cancer was also higher for patients with the comorbidities of benign breast tumor (adjusted HR = 2.39, 95% CI = 1.94−2.95). However, individuals that used progesterone demonstrated a decreased risk of breast cancer (0.53 fold) compared to those that did not use progesterone (95% CI = 0.36−0.77).

### Factors affecting breast cancer risk in patients with or without uterine leiomyoma

We further analyzed factors like age, mammogram screening, comorbidity, and medication status among the breast cancer patients with or without uterine leiomyoma (Table 3). We observed that patients in the uterine leiomyoma group were at a higher risk of breast cancer than those in the leiomyoma-free group, particularly in the age group ≥ 45 years (adjusted HR = 1.45, 95% CI = 1.16−1.80; Table 3). Further, compared to individuals without uterine leiomyoma, patients with uterine leiomyoma patients without comorbidity had a 1.22 fold risk of breast cancer (95% CI = 1.00−1.49) and uterine leiomyoma patients with comorbidity had a 1.57 fold increased risk of breast cancer (95% CI = 1.25−1.98). Non-estrogen users in the uterine leiomyoma group had a 1.35-fold increased risk of breast cancer compared to those in the leiomyoma-free group (95% CI = 1.16−1.57). Non-progesterone users in the uterine leiomyoma group had a 1.32-fold increased risk of breast cancer compared to those in the leiomyoma-free group (95% CI = 1.13–1.53).

**Table 3:** Incidences and hazard ratios of breast cancer based on age, mammogram screening, comorbidity, and medication characteristics in patients with or without uterine leiomyoma

		Uterine leiomyoma						
	No			Yes				
Variable	Event	PY	Rate#	Event	PY	Rate#	Crude HR (95% CI)	Adjusted HR† (95% CI)
**Age group**								
20−39	150	211147	0.71	46	54516	0.84	1.18(0.85, 1.64)	1.17(0.83, 1.63)
40−44	251	166935	1.50	82	42543	1.93	1.28(1.00, 1.64)	1.26(0.98, 1.62)
≥ 45	305	199776	1.53	115	50522	2.28	1.49(1.20, 1.85)***	1.45(1.16, 1.80)***
**Mammogram screening**								
No	651	529987	1.23	208	122759	1.69	1.38(1.18, 1.62)***	1.34(1.14, 1.56)***
Yes	55	47871	1.15	35	24821	1.41	1.23(0.80, 1.88)	1.26(0.83, 1.94)
**Comorbidity^‡^**								
No	487	430742	1.13	127	94866	1.34	1.18 (0.97, 1.44)	1.22(1.00, 1.49)*
Yes	219	147116	1.49	116	52715	2.20	1.48 (1.18, 1.85)***	1.57(1.25, 1.98)***
**Medication**								
Estrogen								
No	687	562350	1.22	229	135002	1.70	1.39(1.20, 1.61)***	1.35(1.16, 1.57)***
Yes	19	15508	1.23	14	12578	1.11	0.92(0.46, 1.84)	0.91(0.45, 1.83)
**Progesterone**								
No	690	559916	1.23	232	136075	1.70	1.38(1.19, 1.60)***	1.32(1.13, 1.53)***
Yes	16	17942	0.89	11	11506	0.96	1.09(0.50, 2.34)	1.16(0.54, 2.50)

CI, confidence interval; HR, hazard ratio; PY, person-years;

^#^Incidence rate per 1,000 person-years;

^†^Multivariable analysis including age, comorbidities of hypertension, diabetes, hyperlipidemia, benign breast tumor, menopausal and postmenopausal disorders and infertility, and medication of progesterone;

^‡^Patients with any comorbidity of hypertension, diabetes, hyperlipidemia, tobacco use disorders, alcohol-related diseases, benign breast tumor, menopausal and postmenopausal disorders, obesity, and infertility were classified as the comorbidity group;

*p < 0.05, ***p < 0.001.

Furthermore, we estimated hormonal therapy for positive estrogen receptor (ER+) or positive progesterone receptor (PR+), and specific targeted therapy for positive human epidermal growth factor receptor 2 (HER2+) for breast cancer patients with or without uterine leiomyoma (Table 4). Though not statistically significant, breast cancer patients in the uterine leiomyoma group received more hormonal therapy for ER+ or PR+ (69.6%) than in the leiomyoma-free group (64.9%).

**Table 4:** Hormonal^†^ and specific targeted^‡^ therapy for breast cancer patients with and without uterine leiomyoma

Variable	Uterine leiomyoma		*p*-value
	No	Yes	
	N (%)	N (%)	
Hormonal therapy for ER+/PR+	458 (64.9)	169 (69.6)	0.18
Targeted therapy for HER2+	79 (11.2)	17 (7.00)	0.06
Hormonal and targeted therapy for ER+/PR+ and HER2+	44 (6.23)	13 (5.35)	0.62
No above hormonal or targeted therapy	213 (30.2)	70 (28.8)	0.69

ER, estrogen receptor; HER2, human epidermal growth factor receptor 2; PR, progesterone receptor;

Chi-square test;

^†^ Hormonal therapy for ER+/PR+ includes tamoxifen and aromatase inhibitors (anastrozole, exemestane, and letrozole);

^‡^ Targeted therapy for HER2+ includes lapatinib, pertuzumab, and trastuzumab.

### Mortality risk for breast cancer patients with or without uterine leiomyoma

Finally, we analyzed the mortality rates among the breast cancer patients with or without uterine leiomyoma (Table 5). Mortality was lower in patients with uterine leiomyoma (4.12%, mean follow-up time was 3.59 ± 2.70 years) than in patients without uterine leiomyoma (8.78%, mean follow-up time was 3.54 ± 2.67 years). In addition, the risk of mortality was lower for patients with uterine leiomyoma than for those without uterine leiomyoma (adjusted HR = 0.41, 95% CI = 0.17−0.76).

**Table 5:** Mortality rate of breast cancer patients with or without uterine leiomyoma

	Uterine leiomyoma	
	No	Yes
Breast cancer (N)	706	243
Death (N)	62	10
Mortality rate (%)	8.78	4.12
Mean followed time ± SD (years)	3.54 ± 2.67	3.59 ± 2.70
Crude HR (95% CI)	1 (Reference)	0.46 (0.19, 0.85)*
Adjusted HR^†^ (95% CI)	1 (Reference)	0.49 (0.25, 0.97)*

CI, person-years; HR, hazard ratio; N, number; SD, standard deviation;

^†^Multivariable analysis including age, comorbidities of hypertension, diabetes, hyperlipidemia, benign breast tumor, menopausal and postmenopausal disorders and infertility, medication of progesterone, and treatments for breast cancer;

*p < 0.05.

## DISCUSSION

To the best of our knowledge, this is the first Asian population-based cohort study to evaluate breast cancer incidence and mortality in patients with or without uterine leiomyoma. Previously, Wise *et al* analyzed 18,538 patients with uterine leiomyoma and 39,209 individuals without uterine leiomyoma from 1995 to 2013 and showed that incidence of breast cancer was 3.06 per 1,000 individuals in the uterine leiomyoma group and 2.23 per 1,000 individuals in the leiomyoma-free group (incidence rate ratio: 1.00, 95% CI = 0.91−1.08, adjusted for age) [14]. Further, stratification analysis showed positive association between subjects with uterine leiomyoma that were diagnosed at an age < 30 year and the risk of pre-menopausal breast cancer [14]. In our study, the Asian population showed lower incidence of breast cancer (Table 2) that subjects from Western countries suggesting influence of different genetic background, lifestyles, and environmental exposure [19, 20]. In another case-control study, Chuang *et al* investigated 4,884 patients with breast cancer and 19,536 individuals without breast cancer from 2002 to 2010 and identified 311 cases (6.4%) of uterine leiomyoma in the breast cancer group and 903 cases (4.6%) of uterine leiomyoma in the non-breast cancer group (OR: 1.42, 95% CI = 1.24−1.63) [15].

In the present study, we used a cohort study design and enrolled a much younger population with more than 88% of the study participants being less than 50 years old. Consistently, we observed that uterine leiomyoma was associated with a higher risk of breast cancer. One of the highlights of our study was the increased risk of breast cancer in the uterine leiomyoma group compared with the leiomyoma-free group.

Another interesting finding of our study was that breast cancer mortality was lower in patients with uterine leiomyoma compared with those without uterine leiomyoma (mean follow-up time was approximately 3.5 years for both groups). One possible explanation is that the complicated balance of sexual hormone and hormone receptor status plays a critical role in the etiology of both breast cancer and uterine leiomyoma. However, the characteristics of each subtype of breast cancer and prognostic markers that determine drug responsiveness are not well characterized. The hormone receptor-positive breast cancers (HR+) are known to respond better to hormonal therapy. Also, estrogen receptor-positive (ER+) and progesterone receptor-positive (PR+) tumors are often less aggressive, low-grade tumors than tumors that are hormone receptor negative (HR–) [21, 22]. Wise *et al* observed statistically insignificant but slightly higher proportion of ER+ breast cancer cases in the uterine leiomyoma group (51.9%, 600/1156) than in the leiomyoma-free group (47.5%, 532/1120) [14]. Similarly, in this study the difference between the uterine leiomyoma and the leiomyoma-free groups were statistically insignificant (Table 4).

Another possible explanation is that the stage of breast cancer at diagnosis was earlier in patients with uterine leiomyoma than those without uterine leiomyoma. Wise *et al* reported that distribution of *in situ*, stage 1, and other stages of breast cancer were 21.4%, 38.3%, and 40.3%, respectively, in the uterine leiomyoma group and 19.3%, 35.4%, and 45.2%, respectively in the leiomyoma-free group [14]. However, the data were not statistically significant. Due to lack of detailed information regarding the initial stage of breast cancer, we could not corroborate these findings.

The common mechanisms that operate in both uterine leiomyoma and breast cancer are unknown. Both are strongly influenced by sex hormones [23, 24]. Biologically, it is plausible to associate both uterine leiomyoma and breast cancer with hormonal pathways. For instance, estrogen induces growth of mammary epithelial ducts and alveoli and regulates smooth muscle cells of uterine leiomyoma [25, 26]. Progesterone, another steroid sex hormone, contributes to the proliferation of both breast and uterine leiomyoma tissues [27]. Moreover, other genetic factors, environmental exposures, socioeconomic status, shared comorbidities, and lifestyle choices may also contribute to breast cancer in patients with uterine leiomyoma.

The strength of this study is that we used a large population to retrospectively evaluate breast cancer risk in women with or without uterine leiomyoma. Since population-based, prospective cohort studies are costly, we used a suitable and economical alternative of conducting a retrospective cohort study using existing insurance data. The National Health Insurance program has covered more than 99.5% of the Taiwanese population since 2010. Universal coverage reduces barriers to healthcare access for all citizens, regardless of socioeconomic background and/or residential location [28]. In the present study, by using National Health Insurance Research Database, we demonstrated “real-world practice outcomes” wherein uterine leiomyoma, breast cancer, and all comorbidities were diagnosed at all levels of clinics during a medical consultation.

However, there are several limitations in our study that need to be considered. First, the International Classification of Disease, 9^th^ Revision, Clinical Modification (ICD-9-CM) algorithm was used to define uterine leiomyoma, breast cancer, and comorbidities. Therefore, although the diagnosis of breast cancer was accurate because a peer-reviewed technique was used to confirm it, the diagnoses of uterine leiomyoma and comorbidities may have had potential variations as they were performed by clinical physicians. To overcome this limitation, an ad hoc committee has been established by the insurance authority to evaluate the claims data to prevent errors and violations. In addition, we selected only diagnoses that appeared twice or more times within a year to increase the validity and accuracy. Second, National Health Insurance Research Database did not provide detailed information about menstrual history, reproductive history, family history, environmental factors, and smoking or drinking habits, which may be associated with breast cancer risk. Therefore, these factors could not be evaluated. In addition, relevant clinical variables, such as image reports, serum laboratory data, pathology results, and stage of cancer were not available in our study for further analysis [29].

In conclusion, the incidence of breast cancer is significantly higher in patients with uterine leiomyoma than those without uterine leiomyoma. Interestingly, overall mortality from breast cancer is significantly lower in patients with uterine leiomyoma than in those without uterine leiomyoma.

## MATERIALS AND METHODS

### Data source

The National Health Insurance program was initiated in 1995, and covered approximately 24 million people in Taiwan. The claims data in the present study was extracted from the National Health Insurance Research Database of Taiwan. The National Health Research Institutes of Taiwan managed and updated the Longitudinal Health Insurance Research Database 2000, which contained the detailed medical information of 1,000,000 randomly selected people from the 2000 Registry for Beneficiaries of the National Health Insurance Research Database. The diagnostic codes are in the format of the ICD-9-CM. The personal identification number of individuals was de-identified in the database before being provided to the researchers. The study was approved and conducted under the supervision of the Research Ethics Committee of China Medical University and Hospital (CMUH-104-REC2-115).

### Participant selection criteria

**Figure 2:**
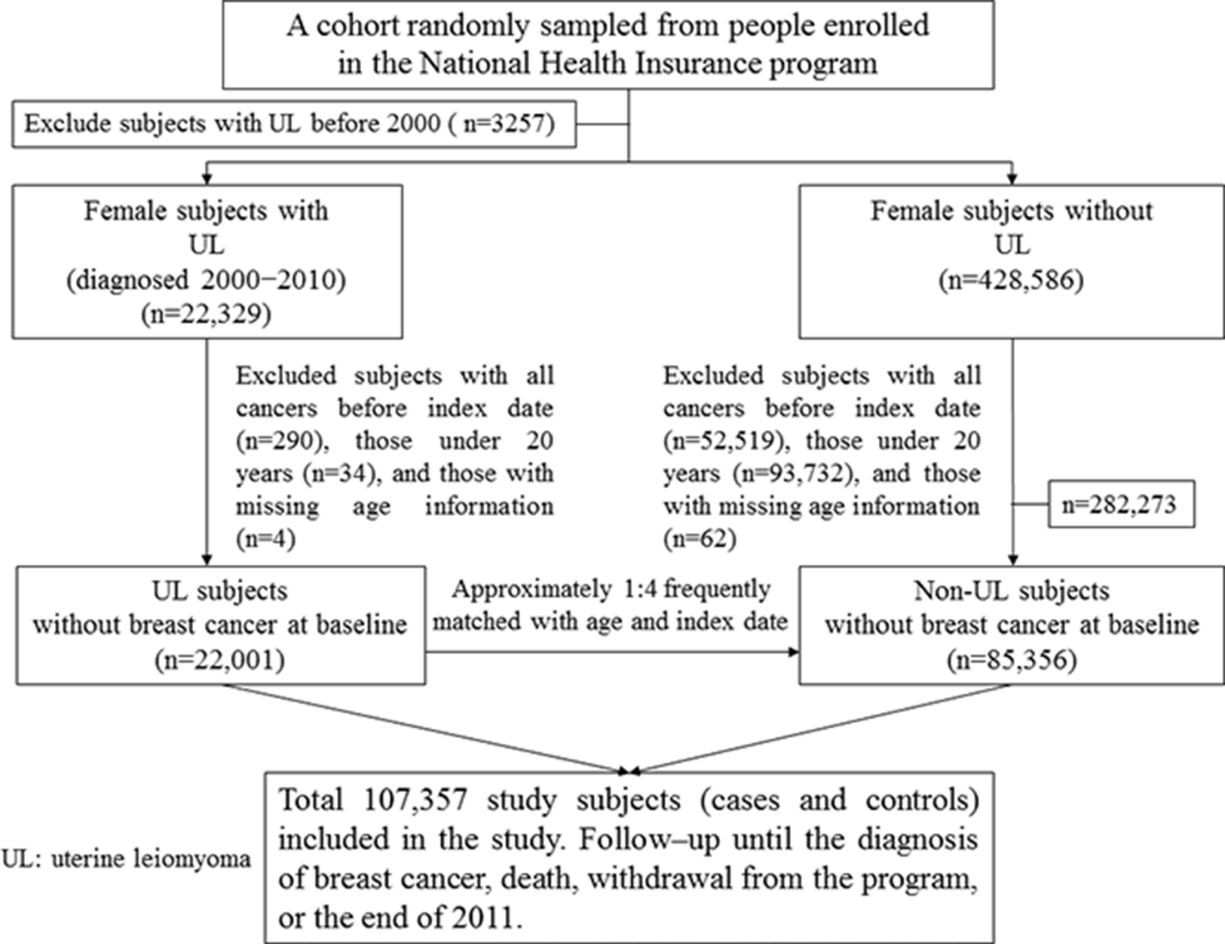
Study design flow chart

The uterine leiomyoma group included patients who were newly diagnosed with uterine leiomyoma (ICD-9-CM code 218) from January 1, 2000 to December 31, 2010. Each uterine leiomyoma patient was coded twice or more within a year, and the first date of coding was defined as the index date. We excluded all patients with a previous history of uterine leiomyoma or any type of cancer (ICD-9-CM codes 140−208) before the index date or those under 20 years of age. For each uterine leiomyoma case, approximately four individuals without uterine leiomyoma matched by age (5-year-range) and index date were randomly selected into the leiomyoma-free group. The exclusion criteria were the same for the uterine leiomyoma and leiomyoma-free groups. Figure 2 shows the selection process of the investigated participants in the uterine leiomyoma and leiomyoma-free groups.

### Outcomes and variables analyzed

The primary endpoint of this study was a diagnosis of breast cancer (ICD-9 code 174) in the registry of catastrophic illnesses. The other endpoints included death, withdrawal from the program, or the end of 2011 (12/31/2011). Times of mammogram screening for breast cancer were calculated until 1 year before cancer diagnosis for breast cancer individuals and until 12/31/2011 for non-breast cancer individuals. The comorbidities that potentially may be related to breast cancer were as follows: hypertension (ICD-9-CM codes 401−405), diabetes mellitus (ICD-9-CM code 250), hyperlipidemia (ICD-9-CM code 272), tobacco use disorders (ICD-9-CM code 305.1), alcohol-related diseases (ICD-9-CM codes 291, 303, 305.0, 571.0–571.3, 790.3, and V11.3), benign breast tumor (ICD-9-CM code 217), menopausal and postmenopausal disorders (ICD-9-CM code 627), obesity (ICD-9-CM code 278), and infertility (ICD-9-CM code 628). We only selected those coded twice or more within a year to confirm the presence of comorbidities. Medications including estrogen and progesterone that may be associated with breast cancer were also evaluated.

### Statistical analysis

Age groups, mammogram screenings, comorbidities, and medications were compared between the uterine leiomyoma and leiomyoma-free groups. The differences were examined using the Chi-square test for categorical variables and the *Student's t-test* for continuous variables. We used the Kaplan-Meier method to estimate the cumulative incidence of breast cancer between the uterine leiomyoma and leiomyoma-free groups and assessed the differences using a log-rank test. The incidence rate of breast cancer was estimated for different risk factors and stratified by age, mammogram screening, comorbidities, and medications in both groups. Univariable and multivariable Cox proportional hazards regression models were used to assess HRs and 95% CIs for the breast cancer risk. Variables in the multivariable model included age, comorbidities of hypertension, diabetes, hyperlipidemia, benign breast tumor, menopausal and postmenopausal disorders, and infertility, and medication of progesterone, which were significantly different in the univariable Cox model. Further data analysis was performed to evaluate hormonal therapy (tamoxifen and aromatase inhibitors like anastrozole, exemestane, letrozole) for ER+ or PR+ and specific targeted therapy (lapatinib, pertuzumab, trastuzumab) for HER2+ breast cancer patients in the uterine leiomyoma group and the leiomyoma-free group. In addition, risk of mortality was evaluated by multivariate analyses including age, comorbidities of hypertension, diabetes, hyperlipidemia, benign breast tumor, menopausal and postmenopausal disorders, and infertility, medication of progesterone, and treatments for breast cancer. Data were analyzed using SAS statistical software for Windows (version 9.4; SAS Institute Inc, Cary, NC), and the two-tailed significance level was set at 0.05.
